# Warts

**Published:** 1886-05

**Authors:** 


### Warts.

Isquierdo has discovered that warts are neoplasms of connective tissue anatomically resembling the sarcomata. He finds large numbers of schizomycetes in the interstices of the fibrous and cellular elements and in the vessels. These schizomycetes are also found in the vessels of the healthy skin about the warts. He believes that these are specific bacteria which circulate in the blood, clogging the vessels of the skin, thus causing, by irritation, the development of the neoplasms. The *bacilli verrucæ* are somewhat larger than those of tuberculosis (from 8 to 20 micromillimeters in length), and have nodosities on their sides.—*Gaz. degli Ospidali.*

